# The artificial sweetener neotame negatively regulates the intestinal epithelium directly through T1R3-signaling and indirectly through pathogenic changes to model gut bacteria

**DOI:** 10.3389/fnut.2024.1366409

**Published:** 2024-04-24

**Authors:** Aparna Shil, Luisa Maria Ladeira Faria, Caray Anne Walker, Havovi Chichger

**Affiliations:** ^1^Department of Botany, Jahangirnagar University, Dhaka, Bangladesh; ^2^Biomedical Research Group, School of Life Science, Anglia Ruskin University, Cambridge, United Kingdom

**Keywords:** artificial sweeteners, neotame, intestinal epithelium, gut, microbiota, nutrition

## Abstract

**Introduction:**

Recent studies have indicated considerable health risks associated with the consumption of artificial sweeteners. Neotame is a relatively new sweetener in the global market however there is still limited data on the impact of neotame on the intestinal epithelium or the commensal microbiota.

**Methods:**

In the present study, we use a model of the intestinal epithelium (Caco-2) and microbiota (*Escherichia coli* and *Enterococcus faecalis*) to investigate how physiologically-relevant exposure of neotame impacts intestinal epithelial cell function, gut bacterial metabolism and pathogenicity, and gut epithelium-microbiota interactions.

**Results:**

Our findings show that neotame causes intestinal epithelial cell apoptosis and death with siRNA knockdown of T1R3 expression significantly attenuating the neotame-induced loss to cell viability. Similarly, neotame exposure results in barrier disruption with enhanced monolayer leak and reduced claudin-3 cell surface expression through a T1R3-dependent pathway. Using the gut bacteria models, *E. coli* and *E. faecalis*, neotame significantly increased biofilm formation and metabolites of *E. coli*, but not *E. faecalis*, reduced Caco-2 cell viability. In co-culture studies, neotame exposure increased adhesion capacity of *E. coli* and *E. faecalis* onto Caco-2 cells and invasion capacity of *E. coli*. Neotame-induced biofilm formation, *E.coli*-specific Caco-2 cell death, adhesion and invasion was identified to be meditated through a taste-dependent pathway.

**Discussion:**

Our study identifies novel pathogenic effects of neotame on the intestinal epithelium or bacteria alone, and in co-cultures to mimic the gut microbiome. These findings demonstrate the need to better understand food additives common in the global market and the molecular mechanisms underlying potential negative health impacts.

## Introduction

Artificial sweeteners have emerged as an essential dietary additive, serving as substitutes for sugar in low-calorie foods and beverages, as well as pharmaceuticals and cosmetics (1, 2). According to global market reports on artificial sweeteners, saccharin, sucralose, aspartame, neotame, acesulfame potassium, and cyclamate are widely accepted artificial sweeteners that have been approved as safe by the US Food and Drug Administration (3). Due to their widespread use, artificial sweeteners have a predicted global market value of USD 3 billion by the end of 2025 (4). Whilst the traditional sweeteners, acesulfame potassium, sucralose, saccharin and aspartame, have been consumed by the public for many years there are more recently developed artificial sweeteners which herald the next generation of sweet additives (5). Neotame is one such new sweetener which was developed in the 1990′s with the commercial benefits of greater sweetness potential and improved stability compared to existing sweeteners (6, 7). Whilst neotame is a non-nutritive additive, it is rapidly metabolized and eliminated with no apparent physiological accumulation in the body (8). Feeding studies with neotame in mice and other test animals did not show adverse physical symptoms, water consumption, or clinical pathology evaluations, therefore the sweetener is considered safe for consumption and was approved by FDA in 2002 and EFSA in 2010(8) The acceptable daily intake (ADI) of neotame is 2 mg/kg body weight per day which is no more than 10 mM per day in an individual with average weight (8) Given the different available forms of neotame, such as agglomerated, encapsulated, co-crystallized with sugar and cyclodextrin complexes, the sweetener is easy and cost-effective to use for food manufacturing and, as such, is found in a range of drinks, sauces, savory and sweet foods, and chewing gums (7, 9). Despite widespread global use of neotame, there are surprisingly few research studies on the biological and physiological effects of the sweetener. Given our emerging knowledge of the health impacts of other artificial sweeteners (10), there is a need to focus studies on the impact of neotame on human health.

Numerous epidemiological studies have highlighted the potential benefits of artificial sweeteners in promoting weight loss and aiding individuals with glucose intolerance and type 2 diabetes mellitus (2), however other research has demonstrated negative health outcomes associated with artificial sweetener consumption (11–13). Of particular relevance in recent studies is the impact of artificial sweeteners on dysbiosis of the gut microbiota. The gut microbiota can play a crucial role in regulation of a range of metabolic, neurological, and immune-related conditions and the link between diet and microbiota is apparent (14–16). It is now well-understood that the artificial sweeteners acesulfame potassium, aspartame, sucralose and saccharin have a significant impact on the presence of certain taxa in the microbiota with increased *Actinobacteria, Bacteroides, Parabacteroides, Staphylococcus and Providencia* phylum noted following exposure to sweeteners (1, 17–19). Worryingly, studies also demonstrate the ability of these sweeteners to cause stress-induced conjugative transfer of antibiotic resistance genes (20, 21). We have previously demonstrated that exposure to acesulfame potassium, aspartame, sucralose and saccharin significantly enhances the pathogenic characteristics of model gut bacteria with a focus on biofilm formation (22). Interestingly, there were differences observed between different sweeteners and bacteria, for example saccharin, sucralose and aspartame induced biofilm formation in *E. coli* whereas in *E. faecalis*, aspartame exposure increased biofilm formation and saccharin and sucralose had no effect (22). As well as the disruptive effects of artificial sweeteners directly on the gut microbiota, our previous studies also showed that model gut bacteria exposed to sucralose and aspartame displayed significant adhesion to and invasion of mammalian gut epithelial cells. This pathogenic profile was accompanied by increased epithelial cell death. Worryingly, our previous studies also noted sweetener-induced breakdown of the intestinal epithelium, in the absence of microbiota, associated with oxidative stress, increased permeability and dysregulated claudin expression at the epithelial cell junctions, specifically reduced claudin-3 levels (23). We further identified the role of the sweet taste receptor, a G-protein coupled receptor called T1R3, in mediating the negative effect of sweeteners on the intestinal epithelium. Indeed, studies showed that inhibition of the sweet taste sensing, either through siRNA knockdown of T1R3 or through exposure to the pan-taste inhibitor, zinc sulfate, significantly attenuated any negative effects of traditional artificial sweeteners on both bacteria and intestinal epithelial cells (22–24). Whilst our studies and others demonstrate the potential negative impact of artificial sweeteners on the gut epithelium and microbiota, these focus on traditional artificial sweeteners such as saccharin, sucralose, aspartame and acesulfame potassium. Given the relatively recent development of neotame, there are limited such studies performed on this sweetener. *In vitro* studies using a bioluminescent bacterial panel indicate some toxicity of neotame which is strain-dependent (25). *In vivo* studies provide more detail and demonstrate that long-term neotame feeding in mice, over 4 weeks, reduces and alters α-diversity and β-diversity respectively in the microbiome. Interestingly, this study showed that neotame-induced metabolic changes in the microbiota with elevated levels of fatty acids and cholesterol and decreased levels of metabolites, such as malic acid, mannose-6-phosphate and glyceric acid, in fecal samples (26). This is further indicative of a changing microbiome profile following neotame exposure and a subsequent negative effect on host ability to absorb fatty acids and lipids. These studies with neotame indicate that the newer synthetic sweetener has potential negative effects on the microbiota but provide limited information on the changes at a bacteria-specific level. Therefore, research is needed to better understand how neotame impacts gut bacteria and how they interact with the intestinal epithelium. In the present study, we utilize two model gut bacteria which are predominantly identified in the microbiota and a human cell model of the intestinal epithelium to investigate this area of research.

## Materials and methods

### Materials

*Enterococcus faecalis* (*E. faecalis*, 19433™) and *Escherichia coli (E. coli*, 10418) were purchased from ATCC (Middlesex, UK) and NCTC (Salisbury, UK), respectively. Field isolates of *Shigella* spp., *Escherichia coli* ESBL producer, *Enterococcus faecalis* and *Enterococcus faecium* were collected, as previously described (27), from bird feces in the Cambridge area. Bacterial media and blood agar plates was purchased from Oxoid (ThermoFisher, Hampshire, UK). Silencing RNA (siRNA) for T1R3 and a DharmaFECT™ reagent were obtained from Dharmacon (Cambridge, UK). Antibodies directed against claudin 3 were purchased from Abcam (Cambridge, UK), while T1R3 and actin antibodies were obtained from Santa Cruz Biotechnology (Santa Cruz, CA). An annexin V kit was purchased from BD Pharmingen (Wokingham, UK). For bacterial growth curve experiment and biofilm assay, sterile, flat-bottom, non-treated polystyrene 96-well plates were purchased from CytoOne (StarLabs, Milton Keynes, UK). Phosphate Buffered Saline (PBS) was obtained from Gibco (ThermoFisher, Hampshire, UK). All other reagents, including human colon carcinoma cell line, Caco-2, and neotame were purchased from Sigma-Aldrich (Dorset, UK).

### Bacterial and mammalian cell culture

Bacterial cells were grown aseptically at 37°C on solid media for single colonies, or in liquid media with shaking (150 rpm) for growth measurements. *E. faecalis* and *E. coli*, were propagated using brain heart infusion (BHI) agar/broth and nutrient agar/broth respectively. Human colon carcinoma cells (Caco-2) were cultured in Eagle's Minimum Essential Media containing 10% fetal bovine serum and 1% penicillin/streptomycin (1 U/mL penicillin, 1 μg/mL streptomycin), and used between passages 35 and 50.

### Mammalian cell viability and apoptosis measurement

Differentiated Caco-2 cells were grown to 60% confluence in T-25 flasks prior to exposure with neotame (0.01 μM to 10 mM) for 24 h. Integrity of the Caco-2 cell monolayer was validated on Transwell inserts using transepithelial electrical resistance (TER) (EVOM^2^; World Precision Instruments, Herts, UK). Resistance higher than or equal to 800 Ω.cm^2^ was considered appropriate for experiments (28). Neotame was dissolved in the vehicle control (H_2_O) and sterile filtered to prepare a working stock solution. For cell viability assays, neotame-treated cells were incubated with MTT reagent (3-(4,5-Dimethylthiazol-2-yl)-2,5-Diphenyltetrazolium Bromide) for 2 h at 37°C. Absorbance was then assessed at 450 nm using a microplate reader (Tecan Sunrise). For apoptosis and cell death assays, adherent and floating cells were collected and incubated with a binding buffer, annexin V, and propidium iodide for 15 min in the dark. Cells were then analyzed with an Accuri C6 Flow cytometer (BD Biosciences), and the percentage of positive cells for annexin V and propidium iodide was calculated with FlowJo (V10.2, Oregon, USA).

### siRNA transfections in mammalian cells

Caco-2 cells were transiently transfected with siRNA specific to T1R3 or non-specific control siRNA using a DharmaFect™ 2 reagent, as per manufacturer's guidelines. Cells were transfected at a seeding density of 0.5 × 10^4^ cells per well of a 96-well plate, 2.5 × 10^4^ cells per well of a Transwell insert, or 1.5 × 10^5^ cells per well of a 6-well plate. Transfected cells were plated onto Transwell inserts or 96-well plates for an analysis of permeability and whole-cell ELISA, respectively. At 24 h post-transfection, cells were exposed to neotame (0.01 μM to 10 mM) or vehicle control (H_2_O) for a further 24 h. Experiments were then performed as outlined in ‘*Whole cell ELISA and epithelial monolayer permeability in mammalian cells*.' To confirm knockdown of T1R3 at 48 h post-transfection, cells were lysed with a radioimmunoprecipitation assay buffer, resuspended in a Laemmli buffer, and subjected to immunoblot analysis. Immunoblot analyses were performed on 10% SDS-PAGE using a primary antibody specific to T1R3 (ab150525) and β-actin (AM4302) at a dilution of 1:1,000 and secondary antibody dilutions of 1:5000. Image J software (version 1.52d) was used to quantify Western blots and T1R3 expression was normalized to actin expression.

### Whole cell ELISA and epithelial monolayer permeability in mammalian cells

Caco-2 cells (1 × 10^4^ cells per well) were plated on black-walled 96-well plate for 24 h, followed by exposure to neotame (0.01 μM to 10 mM), or the vehicle control (H_2_O) for a further 24 h. Where stated, cells were first transfected with siRNA for 24 h, treated with neotame, and then rinsed once with Dulbecco's phosphate-buffered saline (DPBS) and fixed using 1% paraformaldehyde at room temperature for 10 min. Whole cell ELISA was performed as previously described (29) in non-permeabilized Caco-2 cells using antibodies specific to claudin 3 or T1R3 or IgG control. Fluorescent-conjugated secondary antibodies were measured at a 1 s exposure time using a florescent plate reader (Victor, Perkin Elmer), and measurements from blank wells (no primary antibody) were subtracted to provide the presented data. To confirm changes in claudin 3, cells were lysed with a radioimmunoprecipitation assay buffer, resuspended in a Laemmli buffer, and subjected to immunoblot analysis. Immunoblot analyses were performed on 10% SDS-PAGE using a primary antibody specific to claudin 3 (ab214487) and β-actin (AM4302) at a dilution of 1:1,000 and secondary antibody dilutions of 1:5,000. Image J software (version 1.52d) was used to quantify Western blots and claudin 3 expression was normalized to actin expression.

Epithelial monolayer permeability was assessed using the fluorescein isothiocyanate (FITC)-dextran permeability assay. Caco-2 cells were plated onto Transwell filters for 24 h, followed by exposure to neotame (0.01 μM to 10 mM), or the vehicle control (H_2_O) for a further 24 h. Where stated, cells were first transfected with siRNA. Permeability was measured by adding FITC-conjugated to 20 kDa dextran (FD20) to media in the upper chamber of the Transwell filter to a concentration of 5 μg/μl. FD20 was allowed to equilibrate for 180 s at 37°C, and a sample (100 μl) of media from the lower chamber was collected and analyzed at 488 nm using a Victor^TM^ X3 multiplate reader (Perkin Elmer). Permeability (%) was calculated by fluorescence accumulated in the lower chamber divided by fluorescence in the upper chamber, which was then multiplied by 100.

### Bacterial growth curve determination

A single bacterial colony of *E. coli or E. faecalis* was inoculated aseptically into nutrient broth or BHI broth, respectively, supplemented with neotame at a range of concentrations from 0.1 to 1,000 μM, or vehicle [double-distilled water (ddH_2_O)] and allowed to grow for up to 4 days. Growth was recorded as absorbance at 600 nm (A600) using Victor^TM^ X3 multiplate reader (Perkin Elmer).and values were normalized to 0 μM at 0 h (as 1). In addition, *E. coli* (MG1655), *Shigella* spp., *E. coli* ESBL producer, *E. faecium* were used in the investigation. Bacteria were grown for 24 h, at 37°C in Luria-Bertani (LB) broth supplemented with neotame at a range of concentrations from 2 mM to 2 μM, or vehicle (ddH_2_O). Following incubation, the absorbance was read at 620 nm using a spectrophotometer (Tecan'Sunrise).

### Biofilm formation assay

Biofilm formation of *E. coli* and *E. faecalis* was measured after exposure to neotame (100 μM) using the indirect crystal violet biofilm formation assay as described previously (22) with some modifications. In addition, *E. coli* MG1655 and field isolates of *Shigella* spp. and *Enterococcus faecium* were assessed. Bacterial cultures were propagated in LB broth with neotame (1mM) and also in LB broth with sterilized ddH_2_O (vehicle). A single bacterial colony was inoculated into 10 ml of the corresponding liquid media supplemented with sweetener or vehicle (H_2_O) in presence or absence of zinc sulfate. Absorbance at 600 nm was measured on Victor^TM^ X3 fluorescent plate reader (Perkin Elmer) to ensure equal bacterial cell numbers, and the overnight culture was transferred into liquid media (1:200) supplemented with artificial sweeteners. After vortexing, 200 μL was transferred into sterile 96-well plasticware plates and grown aerobically for 48 h at 37°C. The supernatant was removed, and wells were washed twice with ddH2O to remove loosely associated bacteria. Each well was stained with 150 μL 0.1% Gram crystal violet for 20 min at room temperature. After staining, wells were washed with ddH_2_O three times. The retained crystal violet was solubilised by adding 200 μL 30% acetic acid and incubating at 37°C for 5 min. The quantitative analysis of biofilm formation was performed by measuring absorbance at 600 nm using Victor^TM^ X3 fluorescent plate reader (Perkin Elmer). The biofilm forming units were calculated by dividing the absorbance of crystal violet retained with the absorbance of the total bacterial growth and was normalized to the control (as 1).

### Bacterial adhesion assay

Adhesion of the model gut bacteria to Caco-2 cells following artificial sweetener exposure was measured as previously described (22) with some modifications. Caco-2 cells were seeded on 24-well tissue culture plates (7.5 × 10^4^ cells/well) and incubated in humidified condition (90%) at 37°C and 5% CO_2_ for 48 h, following exposure to artificial sweeteners for 24 h. Meanwhile, a single colony of *E. coli* and *E. faecalis* was inoculated into respective media supplemented with neotame (100 μM) in the presence or absence of zinc sulfate (100 μM), or vehicle (ddH_2_O) and incubated overnight at 37°C with shaking at 150 rpm. Bacteria were then washed twice with 500 μL serum and antibiotic-free EMEM media by centrifuging at 4,000 rpm (2683 × *g*) for 10 min at 37°C (accuSpinTM 1R, Fisher Scientific, Thermo Electron Corporation LED GmbH, Osterode, Germany) and re-suspended in EMEM without antibiotics. Caco-2 cell monolayers were washed twice with 500 μl PBS, and then EMEM (490 μL; without antibiotics) was added to each well. The total number of adherent Caco-2 cells was measured by performing a cell count. Bacterial suspension (10 μL) was added on the Caco-2 cells at a multiplicity of infection (MOI) 1:300 for an infection incubation time of 1 h. After the infection period, the cells were washed twice with 500 μL of sterile PBS and the Caco-2 cells were lysed with 500 μL of 0.5% Triton X-100. The number of viable bacteria was determined by spread-plating serial dilutions of the cell suspension on respective solid media, followed by overnight incubation at 37°C and then counting colony forming units. Bacterial adhesion was expressed as ratio of total bacteria attached per viable Caco-2 cells (normalized to 100). Each assay was performed in triplicate with the successive passage of Caco-2 cells.

### Bacterial invasion assay

The ability of bacterial to invade Caco-2 cells was measured as previously described (22). Briefly, Caco-2 cells were seeded on 24-well tissue culture plates for 36 h followed by exposure to neotame for a further 24 h. The cell monolayer was rinsed with sterile PBS and antibiotic-free EMEM media was added for the bacterial invasion assay. In parallel, bacteria were exposed to neotame and prepared for infection. The number of adhered Caco-2 cells that were subjected to bacterial infection was determined by performing a cell count. Caco-2 cell monolayer was infected with bacteria at MOI 1:300 for 1 h at 37°C. The monolayer was washed once with 500 μL PBS and fresh cell culture medium (500 μL) was added containing 100 μg/mL gentamicin for *E. coli* and 100 μg/mL gentamicin along with 50 μg/mL ampicillin for *E. faecalis* and incubated at 37°C for 30 min to kill the external-adhered bacteria. The cell monolayer was washed twice with PBS and then lysed with 0.5% Triton X-100 in PBS.

The number of viable colony-forming units were determined by diluting and plating the samples onto solid media and incubating overnight at 37°C. The results were expressed as the ratio of intracellular bacteria compared with the control (normalized to 100). Each assay was performed in triplicate with the successive passage of Caco-2 cells.

### Cytotoxicity assay

The cytotoxic effect of neotame-mediated bacterial metabolites on intestinal epithelial cells was performed following the protocol previously described (22), and cell viability was measured by using the Cell Counting Kit-8 (CCK-8), as per manufacturer's guidelines. Caco-2 cells were grown on 96-well plates (1 × 10^4^ cells/well) and incubated for 48 h at 37°C in humidified condition with 5% CO_2_. Simultaneously, *E. coli* or *E. faecalis* was grown in 10 ml of respective liquid media supplemented with 100 μM of neotame with or without 100 μM zinc sulfate or vehicle for 24 h. The cultures were centrifuged at 4,000 rpm (2,683 × g) for 15 min at 4°C and supernatant was collected, filter-sterilized (0.22 μM membranes; Millipore, USA). 50 μl of the soluble bacterial factors (supernatant) and 50 μl antibiotic-free EMEM was added to the Caco-2 cell monolayer. Cells were incubated for 24 h followed by measurement of cell viability using CCK-8 reagent assessed as absorbance at 450 nm using a microplate reader (Tecan Sunrise^TM^, Switzerland).

### Statistical analysis

All data sets were statistically analyzed using GraphPad Prism (version 7.05). Analysis was performed using either a one-way or two-way ANOVA with Tukey Multiple comparisons *post-hoc* test where relevant. Statistical significance is considered where *p* < 0.05. Data is presented as mean ± standard error mean (S.E.M.) unless otherwise stated and sample size (n number) is included in the figure legend for each study.

## Results

### Neotame causes epithelial cell damage and disruption of the intestinal epithelial monolayer

**Figure 1:**
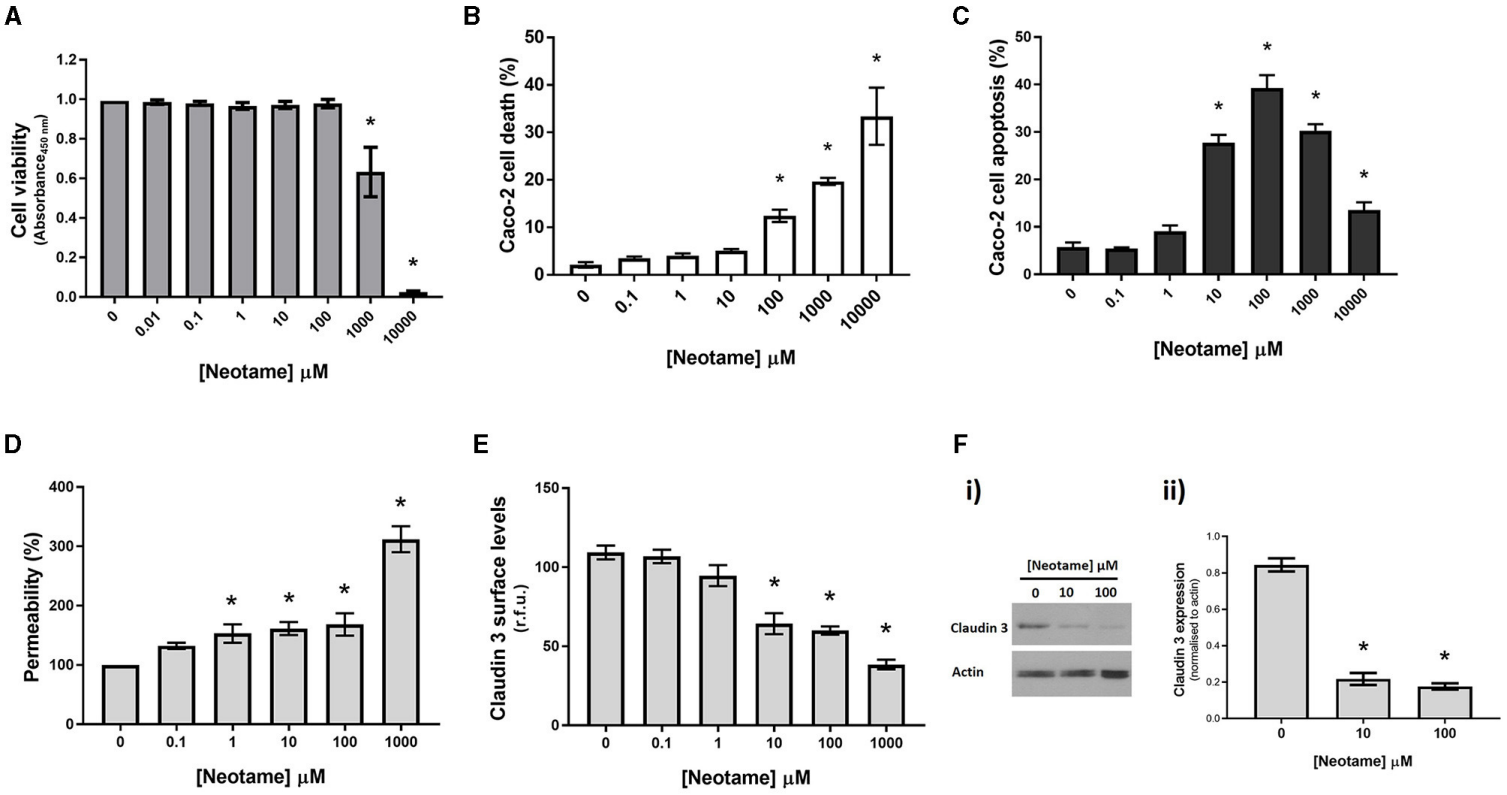
Neotame causes epithelial cell death and disruption of the intestinal epithelial monolayer. Caco-2 cells were exposed to neotame at a range of concentrations for 6 h **(B, C)** and 24 h **(A, D, E, F)**. Cell viability **(A)** was assessed using MTT assay and cell death and apoptosis was assessed using flow cytometry **(B, C)**. Epithelial monolayer permeability was determined using FITC-dextran Transwell assay **(D)** and claudin 3 expression at the Caco-2 cell surface was assessed using whole cell ELISA **(E)** and Western blotting with Caco-2 cell lysates **(F)**. Data are presented as mean ± S.E.M, *n* = 6-8. *p < 0.05 vs. vehicle for neotame (0 μM).

Our previous studies have demonstrated that artificial sweeteners, sucralose, saccharin and aspartame, significantly reduce viability of intestinal epithelial cells (23) therefore our first experiments in this study assessed the impact of neotame on the intestinal epithelium *in vitro* at a range of physiological concentrations. Given that the ADI for neotame is approximately equivalent to 10 mM, we used up to this concentration for initial studies (8). There was a significant increase in Caco-2 cell viability at 1,000 μM neotame concentration exposure with higher concentrations showing very little cell viability (0.025 ± 0.005 a.u. for 10 mM as compared to 0.993 ± 0.042 a.u for 0 mM control) (Figure 1A). These findings were mirrored by cell death studies which noted a significant increase in cell death from 100 μM and higher (Figure 1B) and significant apoptosis of Caco-2 cells from 10 μM neotame exposure and higher (Figure 1C). Given the excessive cell death noted at 10 mM neotame concentration, further studies were performed up to 1,000 μM only. Permeability of the epithelial cell monolayer showed a significant increase at 1 μM neotame and higher (Figure 1D). Whilst increased monolayer permeability at 100 μM and higher could be reasonably expected since Caco-2 cell death would result in leak across the monolayer, findings at 1 μM and 10 μM neotame suggest increased leak due to paracellular junction breakdown. Indeed, whole cell and cell surface expression of the tight junction molecule, Claudin 3, was significantly decreased at 10 μM and higher in Caco-2 cells (Figures 1E, F). Taken together, these data demonstrate the neotame causes intestinal epithelial cell death at high concentrations (100 μM and higher) and leak across the epithelial monolayer at lower concentrations (1–100 μM).

### Neotame regulates Caco-2 cell viability and intestinal epithelial barrier function through T1R3-dependent signaling

**Figure 2:**
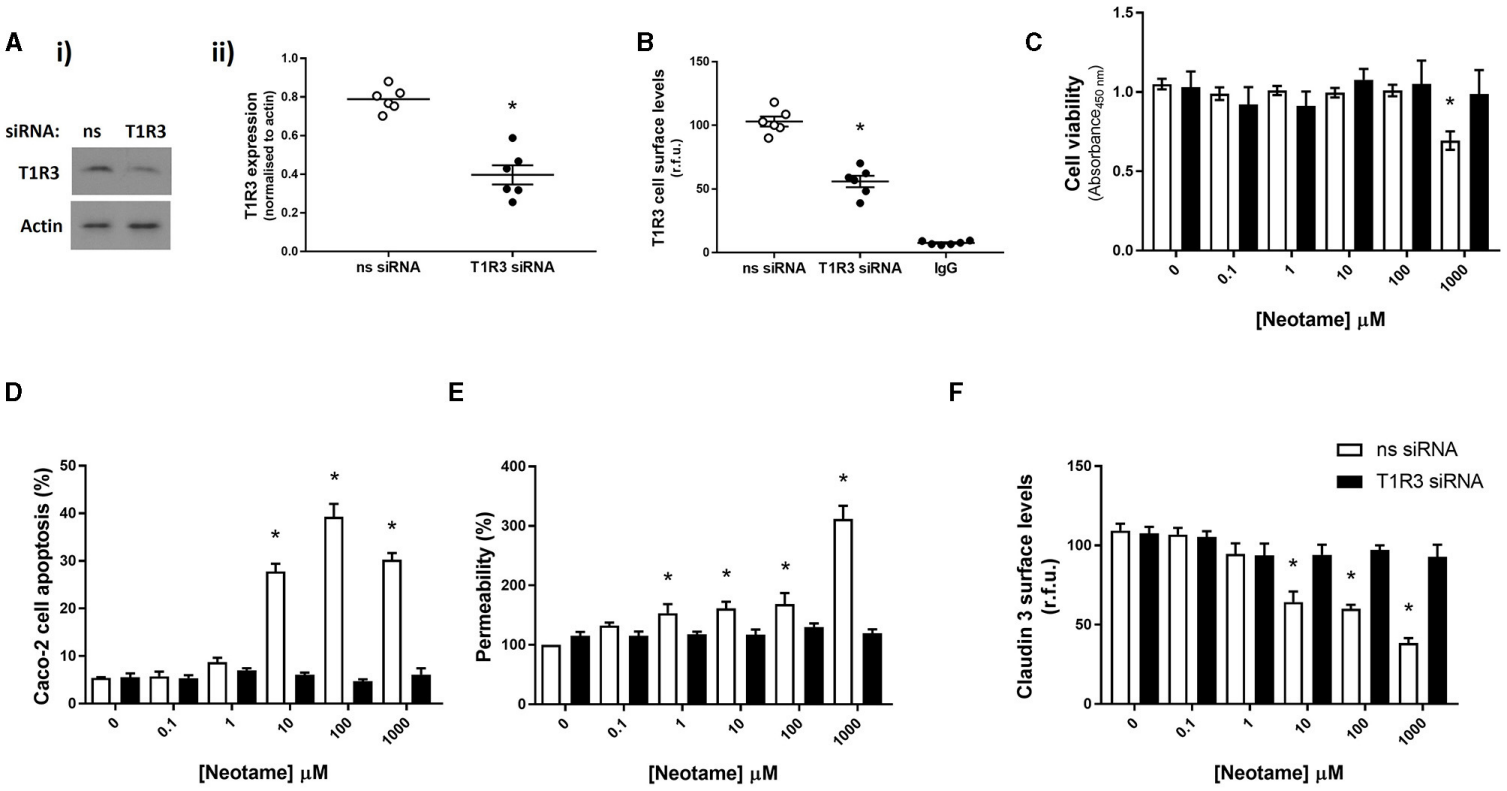
Neotame regulates Caco-2 cell viability and intestinal epithelial barrier function through T1R3-dependent signaling. T1R3 expression was silenced in Caco-2 cells using T1R3-specific siRNA and compared to non-specific (ns) siRNA. Knockdown of protein levels was confirmed using Western blotting, representative blot [**(A)**i] and quantification [**(A)**ii] shown, and whole cell ELISA **(B)**. Following siRNA transfection, Caco-2 cells were exposed to neotame at a range of concentrations for 6 h **(D)** and 24 h **(C, E <** **F)**. Cell viability **(C)** was assessed using MTT assay and cell apoptosis **(D)** was assessed using flow cytometry. Epithelial monolayer permeability **(E)** was determined using FITC-dextran Transwell assay and claudin 3 expression **(F)** at the Caco-2 cell surface was assessed using whole Cell ELISA. Data are presented as mean ± S.E.M, *n* = 6–8. *p < 0.05 vs. non-specific siRNA, vehicle for neotame (0 μM).

We next sought to establish whether this is a direct effect of neotame on sweet taste receptors in intestinal epithelial cells, rather than an indirect chemical effect of neotame. As we have previously demonstrated the presence of the sweet taste receptor T1R3, but not T1R2, in intestinal epithelial cells (23), we investigated Caco-2 cell viability, apoptosis and leak in cells transiently transfected with siRNA specific to the human sweet taste receptor, T1R3. siRNA knockdown of T1R3 expression was confirmed using Western blot [Figures 2A (i), (ii)] and whole cell ELISA (Figure 2B) with both techniques showing a significant decrease in T1R3 expression. Knockdown of T1R3 attenuated the cytotoxic (Figure 2C) and pro-apoptotic (Figure 2D) effects of neotame, as well as the increased monolayer permeability (Figure 2E) and reduced Claudin 3 expression observed at Caco-2 cell surface (Figure 2F). These data demonstrate that neotame-induced damage to the intestinal epithelium *in vitro*, both barrier disruption and cell death via apoptosis, is mediated by the sweet taste receptor, T1R3.

### Exposure to neotame significantly increases biofilm formation by *E. coli* and *E. faecalis*, and cytotoxicity by *E. coli* only, in a zinc-dependent manner

**Figure 3:**
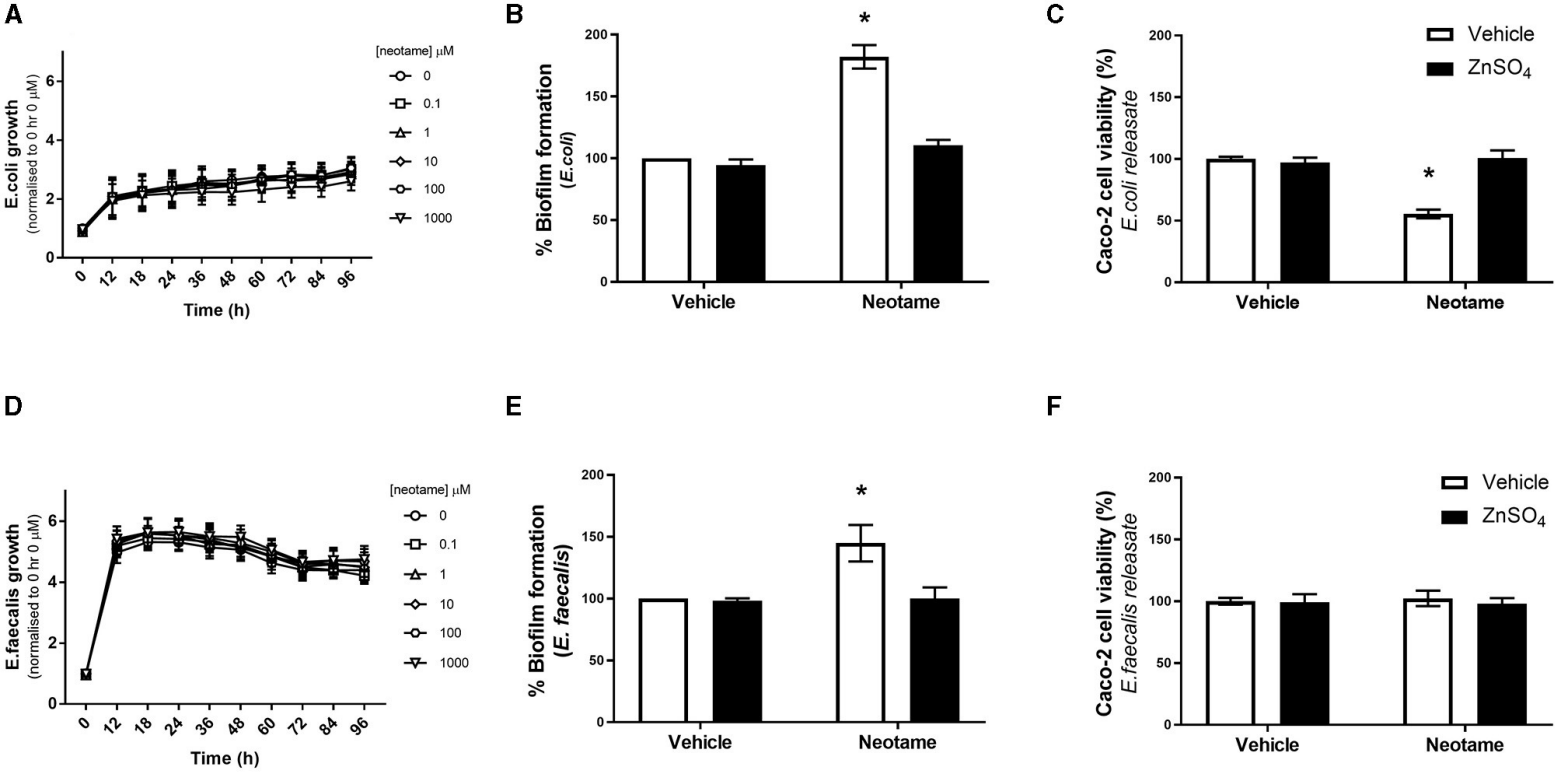
Exposure to neotame significantly increases biofilm formation by *E. coli* and *E. faecalis*, and cytotoxic effect of soluble bacteria factors released from *E. coli*, in a zinc-dependent manner. Bacterial growth of *E. coli* **(A)** and *E. faecalis* **(D)** was measured over 96 h following exposure to neotame at a range of concentrations. Absorbance was measured at 600 nm and normalized to vehicle at 0 h. Biofilm formation of *E. coli* **(B)** and *E. faecalis* **(E)** was measured, using crystal violet assay, following exposure to neotame (100 μM) in the presence and absence of zinc sulfate (100 μM) for 24 h. Cytotoxicity in Caco-2 cells was measured following 24 h exposure to bacterial supernatant, where *E. coli* **(C)** and *E. faecalis* **(F)** were incubated with neotame (100 μM) in the presence and absence of zinc sulfate (100 μM) for 24 h. Data was normalized to vehicle for neotame and presented as mean ± S.E.M, *n* = 6–8. *p < 0.05 vs. vehicle for neotame and zinc sulfate (0 μM).

In physiological settings, the intestinal epithelium is in close association with the gut microbiota and therefore any dietary substances which impact the microbiota will also impact the epithelial barrier. Of note, biofilm formation of gut bacteria significantly disrupts the integrity of the intestinal epithelial monolayer through mechanical force exertion from the biofilm as well as the release of bacterial factors when in a biofilm (30). We have previously demonstrated that the artificial sweeteners, saccharin, sucralose and aspartame, significantly increase biofilm formation in model gut microbiota bacteria, *E. coli* NCTC and *E. faecalis* (22). Therefore, our next studies sought to understand the effect of neotame on these model bacteria. We first investigated whether neotame had an impact on planktonic bacterial growth of *E. coli* NCT and *E. faecalis* and noted no significant change at a range of concentrations, 0.1–1,000 μM, and timepoints up to 96 h (Figures 3A, D). These studies were confirmed in other model gut bacteria (Table 1) demonstrating a robust absence of sweetener-induced effect on bacteria growth across different species. We next sought to investigate the impact of neotame on biofilm formation of model gut bacteria, *E. coli* and *E. faecalis*. Neotame exposure, at 100 μM, significantly increased biofilm formation in both bacteria (Figures 3B, E). The pan sweet taste inhibitor, zinc sulfate, was used to investigate the role of sweet taste sensing in regulating this pathogenic effect (22, 24). Interestingly, zinc sulfate exposure with neotame significantly blocked the increase in biofilm formation observed in both bacteria (Figures 3B, E).

**Table 1:** Neotame does not impact planktonic growth of different model gut microbiota bacterial species.

**Bacterial species**	**Bacterial growth (normalized to vehicle treatment)**				
	**0** μ**M**	**15** μ**M**	**30** μ**M**	**125** μ**M**	**500** μ**M**
*Shigella*	1.000 ± 0.011	0.881 ± 0.015	0.890 ± 0.013	0.872 ± 0.016	0.865 ± 0.016
*E. coli ESBL*	1.000 ± 0.075	0.920 ± 0.069	0.933 ± 0.060	0.942 ± 0.054	0.923 ± 0.040
*E. coli MG1655*	1.000 ± 0.099	0.945 ± 0.124	0.952 ± 0.110	0.941 ± 0.118	0.949 ± 0.121
*E. faecium*	1.000 ± 0.225	0.974 ± 0.206	0.903 ± 0.189	0.891 ± 0.204	0.900 ± 0.215

Bacteria were exposed to neotame at a range of concentrations for 24 h and growth was measured as O.D. and normalized to vehicle treatment (0 μM). Data are presented as mean ± S.E.M, n = 3.

The release of soluble factors from bacteria in a biofilm is associated with pathogenic effects (30). Therefore, we next studied whether neotame induces a change in released bacterial factors which can affect Caco-2 cells. Following 24 h exposure with neotame at 100 μM, solubilised releasate from *E. coli* and *E. faecalis*, called *E. coli*-neotame or -vehicle and *E. faecalis*-neotame or -vehicle, was collected and Caco-2 cells were exposed to each for 24 h. We observed a significant decrease in Caco-2 cell viability following exposure to *E. coli*-neotame compared to *E. coli*-vehicle (Figure 3C). In contract, *E. faecalis*-neotame had no impact on Caco-2 cell viability (Figure 3F). Interestingly, the cytotoxic effect of releasate from *E. coli* exposed to 100 μM neotame on Caco-2 cells (Figure 3C) was significantly higher than the effect of 100 μM neotame alone on Caco-2 cell viability (Figure 1A) (% change for neotame only: 1.41 ± 2.13 vs. % change for E. coli-neotame releasate: 44 ± 4.40, p < 0.05). Furthermore, incubation of *E. coli* with neotame and zinc sulfate blocked the cytotoxic effect of releasate from the bacteria (Figure 3C). Taken together, these data demonstrate the neotame exposure has a significant effect on biofilm formation of *E. coli* and *E. faecalis* through a taste-dependent pathway. Furthermore, neotame also causes *E. coli* to produce soluble factors which result in mammalian cell toxicity through a taste-dependent pathway.

### Neotame significantly disrupts the Caco-2 cell—Bacteria interaction in a zinc-dependent and -independent manner

**Figure 4:**
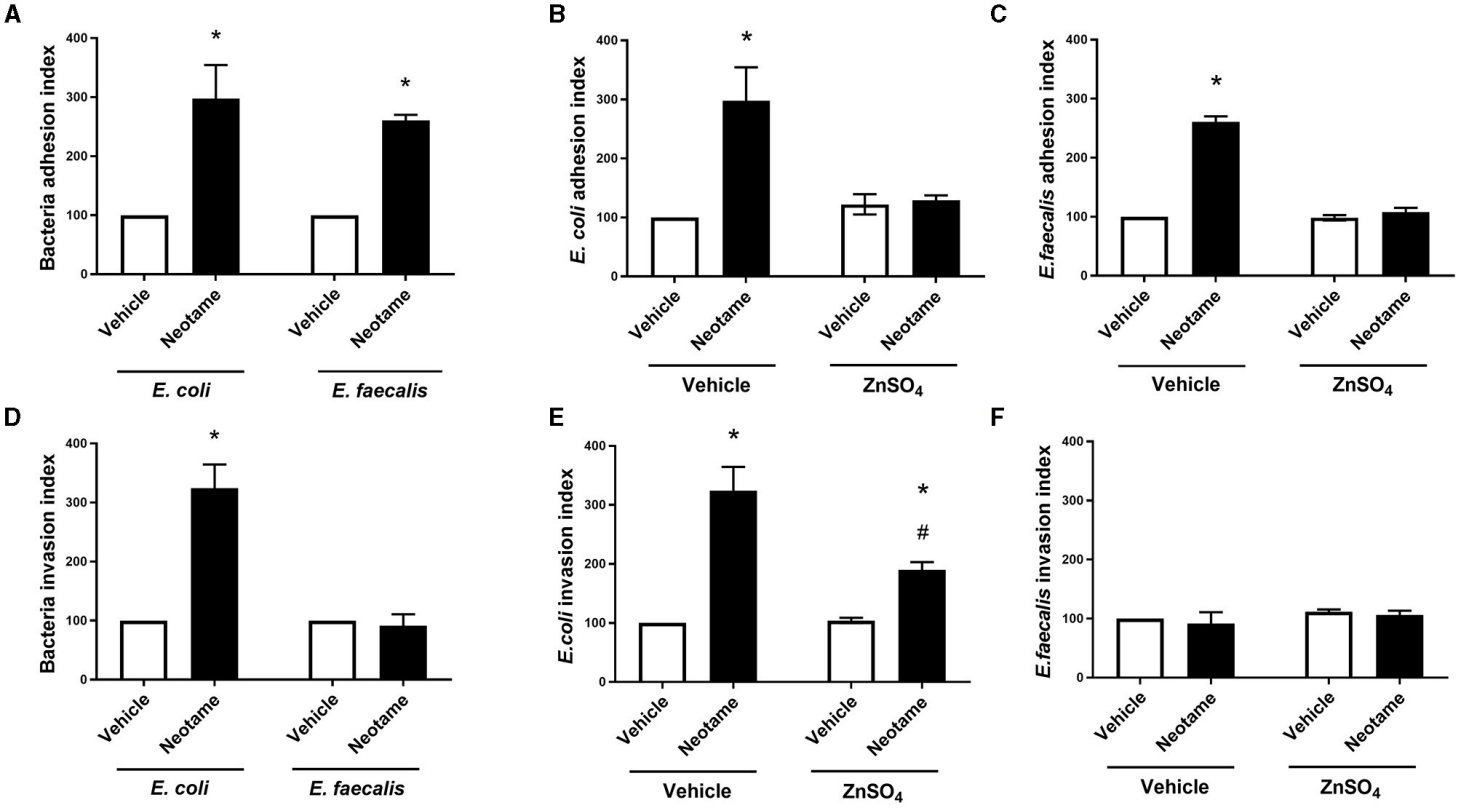
Neotame significant disrupts the Caco-2 cell—bacteria interaction in a zinc-dependent and -independent manner. Bacteria cell adhesion to **(A–C)** and invasion of **(D–F)** Caco-2 cells was measured following *E. coli* **(A, B, D, E)** and *E. faecalis* **(A, C, D, F)** exposure to neotame (100 μM) in the presence and absence of zinc sulfate (100 μM). Bacteria adhesion index is expressed as ratio of total bacteria attached per viable Caco-2 cells (normalized to 100) and bacteria invasion index is expressed as the ratio of intracellular bacteria compared with the control (normalized to 100). Data is presented as mean ± S.E.M, *n* = 5–6. *p < 0.05 vs. vehicle for neotame and zinc sulfate (0 μM), ^#^*p* < 0.05 vs. vehicle for zinc sulfate only.

Bacterial adhesion to and invasion of intestinal epithelial cells represent the initial phases of pathogenic characteristics in many disorders. Therefore, we next investigated the effect of neotame exposure on the adhesive and invasive capability of model gut bacteria with Caco-2 cells. Both *E. coli* and *E. faecalis* treated with 100 μM neotame displayed significantly higher adhesion to Caco-2 cells (Figure 4A). In the presence of the pan sweet taste inhibitor, zinc sulfate, neotame-induced adhesion of *E. coli* and *E. faecalis* to Caco-2 cells was attenuated (Figures 4B, C). Likewise, exposure to neotame significantly increased *E. coli* invasion but had no effect on the invasive capacity of *E. faecalis* (Figure 4D). Whilst zinc sulfate treatment significantly reduced neotame-induced invasion of *E. coli* into Caco-2 cells, it did not completely abrogate invasion caused by the sweetener (Figure 4E). Unsurprisingly, zinc sulfate had no impact on the invasive capacity of *E. faecalis* (Figure 4F). Taken together, these data demonstrate that neotame significantly increases the pathogenic effect of two model gut bacteria on human intestinal epithelial cells, to different degrees, through a taste-dependent mechanism.

## Discussion

**Figure 5:**
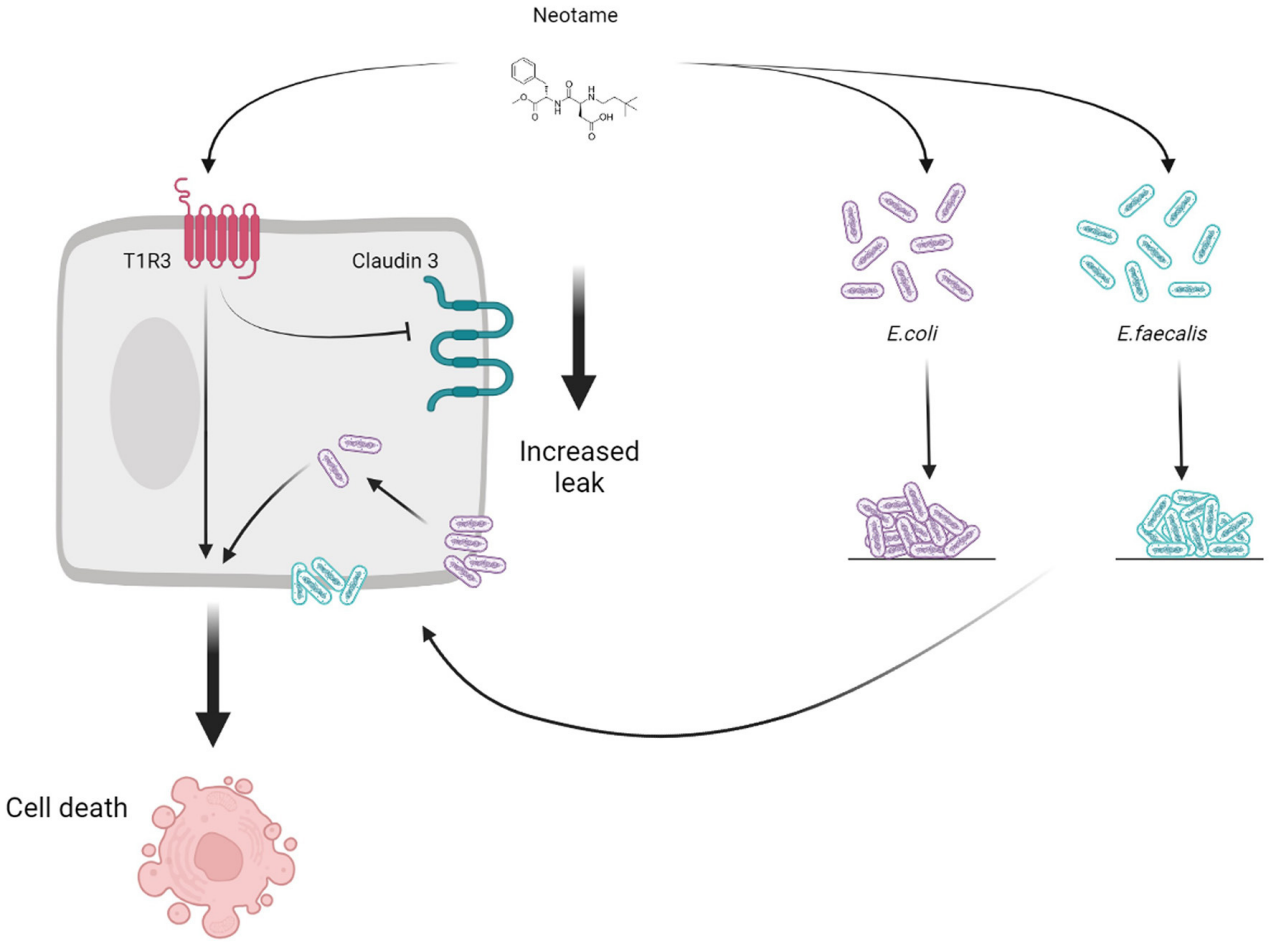
Schematic to show the direct (T1R3-dependent) and indirect (bacteria-dependent) impact of the artificial sweetener neotame on the intestinal epithelial cell.

Artificial sweeteners have historically been regarded as safe additives to enhance the sweet taste profile of a wide range of commercial products however, recent research suggests that certain sweeteners may disrupt the gut microbiota, and thus have a negative effect on host health. In the present study, we investigate the effect of the relatively new synthetic sweetener, neotame, on models of gut bacteria and the human intestinal epithelium. Our findings are the first to demonstrate that neotame can damage the intestinal epithelium directly, through the sweet taste receptor, T1R3, and indirectly, through stimulating pathogenic changes in model gut bacteria which are closely associated with the epithelium (Figure 5). The negative effect of neotame on the epithelium-microbiota relationship in the gut has the potential to influence a range of gut functions resulting in poor gut health which impacts a range of conditions including metabolic and inflammatory diseases, neuropathic pain, and neurological conditions (31–34).

The health impacts of artificial sweeteners have been an area of recent interest with the World Health Organization publishing a 2023 report outlining that these non-nutritive additives should not be used as a weight-control aid (10). This is following a slew of studies which demonstrate the effect of sweeteners on the gut microbiota to influence gastric hormones and glucose intolerance (1, 11). Although the effects of traditional sweeteners on the gut microbiota are well understood, newer sweeteners on the market, such as neotame, have not yet been fully investigated. At nearly double the sweet taste perception of sucralose, neotame is an intensely sweet additive which provides no source of energy and is rapidly metabolized and eliminated (35). As such, it is increasingly used as an artificial sweetener in food production and therefore widely consumed in the diet. In the present study, we investigated the biological effects of neotame on the human intestinal epithelial cell line, Caco-2, and noted cell death, mediated by apoptosis. At concentrations higher than 100 μM we see a switch from pro-apoptotic cells to dead cells suggestive of a toxic effect of neotame. This is similar to previous findings with the artificial sweeteners saccharin and aspartame found to increase cell death in a variety of different cell types including cancer, neuroprogenitor and pancreatic islet cells (23, 36–38). Caco-2 cells are a well-established model of the intestinal epithelium with differentiation resulting in a well-organized brush border and a range of molecular transporters and enzymes expressed to mimic the intestinal epithelium *in vivo* (39). However, these are colon carcinoma cells cultured to mimic the gut milieu, that is, without the humoral, neurological, muscular or immunological elements associated with the gut lumen environment (40). There is thus a need for the use of gut organoid models or *in vivo* feeding studies to investigate the negative impact of neotame on intestinal epithelial cell function, however our studies provide a good indication that this sweetener would significantly disrupt the epithelium in either of these physiological models. In contrast to the human cells, the different model gut bacteria studied in the present work, *E. faecalis, Shigella, E. faecium*, and a range of *E. coli*, pathogenic and non-pathogenic did not show any changes in growth curve in response to neotame exposure at concentrations between 0 and 2 mM. Whilst some studies demonstrate similar outcomes with different bacteria exposed to a range of artificial sweeteners, such as saccharin, aspartame and sucralose (23, 41), studies on multi-drug resistant bacteria such as *Acinetobacter baumannii* and *Pseudomonas aeruginosa* in the presence of sweeteners such as acesulfame potassium, sucralose and saccharin show significant bactericidal effects (42). These differences in the literature may be due to the differences in concentration of sweetener studied. For example, de Rios *et al* studied sweeteners up to 440 μM (42) and Wang *et al* investigated concentrations in the 30–80 mM range, which are significantly higher than the concentrations described to be physiological (43) whereas our studies focused on sweeteners at 100 μM. Hence, the contradictory results from recent research could be explained due to differences in the concentration of the sweeteners used. Another key difference is the use of neotame in the present study, as opposed to previous studies on more traditional artificial sweeteners such as sucralose, saccharin and acesulfame potassium. This highlights the need for further studies on the toxic effects of more recently-developed artificial sweeteners using a range of mammalian and bacterial cell models to map potential health impacts of these additives.

In mammalian cells, the G-protein coupled receptors T1R2 and T1R3 have been established to be sweet taste receptors which responds to sugars and artificial sweeteners in a range of oral and extra-oral locations (44). We have previously identified T1R3 only in the intestinal epithelium (23) and, as such, investigated whether neotame-induced cell death and barrier disruption was mediated through a direct effect on the sweet taste receptor or through a non-specific indirect chemical effect on the intestinal epithelial cells. Following molecular inhibition of T1R3, cell death and apoptosis following exposure with neotame was completely abolished. Likewise, neotame-induced epithelial barrier permeability and claudin 3 internalization was abolished in T1R3-siRNA cells. We have previously identified the pivotal role of T1R3 in mediating epithelial cell damage induced by artificial sweeteners saccharin and aspartame (23) and, whilst not unexpected, there are studies where sweeteners impact cell function independently of the sweet taste receptor (45). Interestingly, bacteria have not been identified to have a homologous sweet taste receptor but our findings here demonstrate the ability of *E. coli* and *E. faecalis* to respond to neotame. Zinc sulfate is a potent but crude inhibitor of sweet taste sensing mediated by T1R3 (24) which we demonstrate to block neotame-induced pathogenic effects in both bacteria. Whilst this supports the notion that there is a type of zinc-sensitive sweet taste sensor in *E. coli* and *E. faecalis*, further studies are needed to identify the specific mechanism through which bacteria can respond to artificial sweeteners. It is possible that sweeteners may induce an oxidative stress response in bacteria, as demonstrated by Yu *et al* with elevated superoxide production in fecal bacteria following exposure to high concentrations of saccharin, sucralose, aspartame or acesulfame potassium (21). Indeed, both ROS- and SOS-related genes are upregulated following exposure to the sweeteners suggesting there may be multiple bacterial sweet taste sensors which can respond to sweetener stimulus (20, 21, 46). In the present study, we identify a range of pathogenic responses elicited by exposure of *E. coli* and *E. faecalis* to neotame, including biofilm formation and increased adhesion to and invasion of mammalian cells. Our studies used laboratory strains of each bacteria, grown individually and in aerobic conditions, as opposed to the gut microbiota setting where over 100 trillion bacteria co-exist in an anaerobic microenvironment (47). Whilst this poses a potential limitation to the studies performed, our research clearly demonstrates that neotame causes pathogenic changes to model bacteria which are associated with a significant risk to human health. For example, the National Institute of Health have linked 60–80% of all microbial infections with biofilm formation (48) and entero-adherent and entero-invasive *E. coli* have been closely aligned to a range of gastrointestinal disorders including diarrhea, intestinal inflammation, and subsequent syndromes (49). Therefore, understanding the impact of neotame on the pathogenic changes occurring in the gut microbiota, and the underlying mechanisms which cause these changes, is vital to understanding how sweeteners impact human health.

Artificial sweeteners are consumed in a range of different food and drink products across the population and therefore it is challenging to assess what are the physiological concentrations of neotame which the intestinal epithelium and microbiota would be exposed to in a standard diet. Previous *in vivo* studies used a range of concentrations of neotame from 0.75 mg/kg body weight in mice to a range of 10–500 mg/kg body weight in pigs (26, 50). The acceptable daily intake in humans is up to 2 mg/kg body weight which, considering the average adult weight and gastric fluid volume is equivalent to 40 mg/L (8, 51, 52). There is little evidence around the accumulation concentration of artificial sweeteners in the intestine however, given known concentrations of sweeteners in commercial products, it is possible that following consumption of a diet soft drink, for example, the intestine could be exposed to up to 2 mM sweetener (2). In the present study, we investigated the effect of neotame at concentrations ranging from 0.1 to 50 mM but noted intestinal epithelial cell death at 0.1 mM and intestinal barrier disruption at 1 μM. Furthermore, co-culture studies with *E. coli* or *E. faecalis* demonstrated pathogenic effects at 100 μM, which is lower than the expected concentration in many food and drink, and the acceptable daily intake (2, 8). It is worth noting, however, that studies were performed following 24 h exposure to neotame whereas transit time in the intestine is 5 h therefore it is possible that the epithelium and gut microbiota would not be exposed to sustained sweetener for as long as was studied (51). Further studies on a range of shorter time points of neotame exposure would therefore provide a more physiological review of the impact of the sweetener on the intestine.

## Data availability statement

The raw data supporting the conclusions of this article will be made available by the authors, without undue reservation.

## Ethics statement

Ethical approval was not required for the studies on humans in accordance with the local legislation and institutional requirements because only commercially available established cell lines were used.

## Author contributions

AS: Writing – review & editing, Writing – original draft, Visualization, Methodology, Investigation, Formal analysis, Data curation, Conceptualization. LL: Writing – review & editing, Methodology, Investigation, Formal analysis. CW: Supervision, Project administration, Writing – review & editing, Methodology. HC: Writing – original draft, Visualization, Investigation, Formal analysis, Data curation, Conceptualization, Writing – review & editing, Supervision, Project administration, Methodology.
